# Chromosomal imbalances in human bladder urothelial carcinoma: similarities and differences between biopsy samples and cancer stem-like cells

**DOI:** 10.1186/1471-2407-14-646

**Published:** 2014-09-01

**Authors:** Donatella Conconi, Elena Panzeri, Serena Redaelli, Giorgio Bovo, Paolo Viganò, Guido Strada, Leda Dalprà, Angela Bentivegna

**Affiliations:** Department of Surgery and Translational Medicine, University of Milan-Bicocca, via Cadore 48, 20052 Monza, Italy; Depatment of Pathology, S. Gerardo Hospital, Monza, Italy; Urology Division, Bassini Icp Hospital, Milano, Italy

**Keywords:** Transitional Cell Carcinoma, Array Comparative Genomic Hybridization, DNA copy number alterations, cancer stem like-cells

## Abstract

**Background:**

The existence of two distinct groups of tumors with different clinical characteristic is a remarkable feature of transitional cell carcinomas (TCCs) of the bladder. More than 70% are low-grade (LG) non-infiltrating (NI) cancers at diagnosis, but 60-80% of them recur at least one time and 10-20% progress in stage and grade. On the other hand, about 20% of tumors show muscle invasion (IN) and have a poor prognosis with <50% survival after 5 years. This study focuses on the complexity of the bladder cancer genome, and for the first time to our knowledge, on the possibility to compare genomic alterations of in vitro selected cancer stem-like cells (CSCs), and their original biopsy in order to identify different genomic signature already present in the early stages of tumorigenesis of LG and HG tumors.

**Methods:**

We initially used conventional chromosome analysis on TCC biopsies with different histotypes (LG vs HG) in order to detect rough differences between them. Then, we performed array comparative genomic hybridization (aCGH) on 10 HG and 10 LG tumors providing an overview of copy number alterations (CNAs). Finally, we made a comparison of the overall CNAs in 16 biopsies and their respective CSCs isolated from them.

**Results:**

Our findings indicate that LG and HG bladder cancer differ with regard to their genomic profile even in the early stage of tumorigenesis; moreover, we identified a subgroup of LG samples with a higher tendency to lose genomic regions which could represent a more aggressive phenotype.

**Conclusions:**

The outcomes not only provide valuable information to deeper studying TCC carcinogenesis, but also could help in the clinic for diagnosis and prognosis of patients who will benefit from a more aggressive therapy.

**Electronic supplementary material:**

The online version of this article (doi:10.1186/1471-2407-14-646) contains supplementary material, which is available to authorized users.

## Background

Bladder cancer is the fourth most common cancer in men and the eighth in women in both incidences and mortality and over 90% of bladder tumors are transitional cell carcinomas (TCCs). The existence of two distinct groups of tumors with different clinical features is a remarkable feature of TCC. More than 70% are in fact low-grade (LG) non-infiltrating (NI) cancers at diagnosis, that can be treated endoscopically by transurethral resection (TUR) alone, recur at least once and 10-20% progress in stage and grade. Thus repetitive and costly follow-up based on urine cytology, cystoscopy and imaging studies of the urinary tract is required, even if the prognosis is usually good. On the other hand, about 20% of tumors show muscle invasion (IN) at diagnosis and have a poor prognosis with <50% survival after 5 years [1]. A model for at least two major pathways has emerged based on the existence of these two distinct groups of lesions [2, 3]. LG NI tumors are generally characterized by constitutive activation of the receptor tyrosine kinase–Ras pathway, and they have activating mutations in the proto-oncogene *FGFR3* [4, 5]; in contrast, inactivating mutations of the tumor suppressor pathways of *TP53*, *RB1* or *PTEN* were found in muscle-invasive lesions [5, 6]. Array comparative genomic hybridization (aCGH) studies have been instrumental in delineating genomic regions that are targeted by copy number changes, called Copy Number Alterations (CNAs). Several aCGH studies of bladder cancer have been published to date and provide the identification of a number of genomic regions of DNA amplification that contain known or candidate oncogenes including cyclin D1 (*CCND1*) on 11q13 [7, 8], *ERBB2* on 17q21 [9], *MDM2* on 12q14–q15 [10], and *E2F3* on 6p22 [11]. Similarly, deletions of genomic regions containing tumor suppressor genes, such as *CDKN2A*, *DBC1* and *TSC1* (at 9p21, 9q33 and 9q34, respectively), *PTEN* on 10q23, *RB1* on 13q14, and *TP53* on 17p13 [12]. Some of these aberrations have been associated with the pathological stage and/or outcome of bladder cancer. Several studies evidenced exclusive genomic alterations in LG and HG tumors [13], with a significant increase in CNAs and genomic instability with increasing stage/grade and with outcome [14, 15]. In addition, the over-representation of focal amplifications, such as at chromosome 6p22, was significantly associated with HG IN tumors [16] and recurrent cases [17].

Since it is currently believed that bladder cancer is derived from a common cancer stem cell (CSC) likely derived by transformation of urothelial cells of the basal layer, bladder CSCs have been isolated based on basal cell markers such as CD44. Nevertheless, in this way, CSCs have only been identified in HG IN tumors [18–21] suggesting that a distinct progenitor cell type exists for LG NI. A recent study of Dancik et al. provides evidence of the existence of distinct progenitor cells in NI and IN tumors, supporting new conceptual framework for investigating and understanding bladder cancer [22]. CSCs are responsible for treatment failure and cancer recurrence since they exhibit specific stem cells features, such as growth as nonadherent spheres in a stem cell medium, unlimited self-renewal, multipotency and lineage-specific differentiation [23]. Understanding the origins and supporting mechanisms of these cells and their relation to the bulk population has a great relevance for improving the knowledge of cancer pathogenesis and therapeutics [23].

In the first step of this study we performed conventional chromosome analysis on TCC biopsies with different histotypes (LG vs HG) in order to detect rough differences between them. We subsequently performed aCGH analysis on another set of 20 biopsies to look for chromosomal imbalances and smaller differences. In a second step, we compared the global pattern of CNAs in 16 of these biopsies with the corresponding isolated CSCs in order to reveal specific genomic aberrations that would provide them with growth advantages and a more aggressive phenotype.

## Methods

### Ethics statement

This study was approved and founded by Direzione Generale Sanità Regione Lombardia and presented by General Director and ethic commitment of ICP Hospital Bassini, Milan. Written informed consent was obtained from the study participants before tissue collection.

### Tumor specimens

45 tumor specimens were obtained by transurethral resection of the bladder (TURB) in a consecutive series of patients newly diagnosed with TCCs at a single center. Staging and grading were done according to the World Health Organization Consensus Classification by a pathologist [24]. They were distinguished in high or low grade (HG or LG) and in muscle invasive or not (IN or NI) (see Additional file 1: Table S1).

### Cells’ isolation

CSCs were isolated from 35 biopsies following a published protocol [25]. Briefly, biopsies were subjected to mechanical and enzymatic digestion and the resulting single cells were cultured in a specific medium with 20 ng/ml epidermal growth factor (EGF) and basic fibroblast growth factor (bFGF) (TebuBio, Rocky Hill, NJ, USA). Cells were seeded at a low density (2×10^4^ viable cells/ml) in the absence of supplementary substrate or adhesion factors and let grow for one week under standard culture conditions.

### Conventional chromosome analysis

Biopsies were subjected to mechanical disruption and incubated for 18-24 h with RPMI-1640 (Euroclone Spa, Milano, Italy) supplemented with 20% FCS. Metaphase chromosome spreads were prepared by direct technique following an overnight colcemid treatment (0.1 μ⁄mL). The fragments were incubated with hypotonic solution with sodium citrate tribasic dihydrate (1%) for 30 minutes at room temperature (RT), while in the same time, eventual cells which were in suspension were pelleted and subjected to hypotonic treatment with 0.56% w/v KCl for 20 minutes at RT. Then cells were fixed with 3:1 methanol:acetic acid. The chromosomes were QFQ-banded using quinacrine mustard and slides were mounted in McIlvaine buffer. The number of metaphases depends on the quality of chromosome preparations. The karyotype was defined following the guidelines of the International System for Chromosome Nomenclature 2009 (ISCN 2009).

### Fluorescence in situ hybridization (FISH)

Fluorescence in situ hybridization was carried out using commercial probes: whole chromosome painting (wcp) probe for chromosome Y (Cytocell, Cambridge, United Kingdom), Vysis SRY Probe LSI SRY Spectrum Orange/CEP X Spectrum Green (Vysis, Abbott Molecular, Abbott Park, Illinois, U.S.A.), or UroVysion bladder cancer kit (Vysis, Wiesbaden, Germany), according to the manufacturer's instructions. The procedures were assessed according to the manufacturer’s protocol and a minimum of 50 nuclei were evaluated. All digital images were captured using a Leitz microscope (Leica DM 5000B) equipped with a charge coupled device (CCD) camera and analyzed by means of Chromowin software (Thesi Imaging, Milano, Italy).

### DNA extraction for array comparative genomic hybridization (aCGH)

Genomic DNA was extracted from fresh biopsies after enzymatic digestion with collagenase H (Roche, Mannheim, Germany). Cells were harvested, washed with a saline solution, digested with proteinase K (Roche, Mannheim, Germany) and purified using phenol/chloroform (Carlo Erba, Milan, Italy). In 16 cases the same procedure for DNA extraction was applied to the isolated CSCs after one week under culture conditions that favor stem cell growth (see above).

### Array comparative genomic hybridization (aCGH) experiments

Sample preparation, slide hybridization, and analysis were performed using SurePrint G3 Human CGH Microarray 8×60K (Agilent, Santa Clara, CA) according to the manufacturer’s instructions. Sex-matched commercial DNA samples (Promega) were used as reference DNA during aCGH. The arrays were scanned at 2-mm resolution using Agilent microarray scanner and analyzed using Feature Extraction v10.7 and Agilent Genomic Workbench v5.0 softwares. The Aberration Detection Method 2 (ADM2) algorithm prompted by Genomic Workbench software was used to compute and assist the identification of aberrations for a given sample (threshold = 5; log2 ratio = 0.3). To calculate the estimated percentage of mosaicism we used the formula determined by Cheung et al. [26].

### Gene ontology analysis

To analyze which ontology classes were over- and under-represented among the genes contained in gain and loss regions detected by aCGH in both biopsies and cancer stem-like cells, we used the GOstat software [27]. The GO terms in the output are linked to a visualization tool for the GO hierarchy (AmiGO, the Gene Ontology database, version 1.8).

### Characteristics of the analyzed samples

In this study we collected 45 primary TCCs, six females and 39 males. The mean age was 73.88 [SD = 12.9] years. The tumors were low grade in 26 patients, and only one case was infiltrating; 19 were high grade, 13 of which were infiltrating (see Additional file 1: Table S1). We were able to establish cultures of CSCs from 35 biopsies applying our published protocol for the isolation and characterization of CSCs from Bladder Cancer [25], because in 10 cases the tumor was of inadequate size for processing, as specimens from TURB are generally very small (less than 50% exceed one cm) [25]. Furthermore, as the number of isolated cells was too low, in 19 cases the cultures have died within a week. Additional file 1: Table S1 and Additional file 2: Figure S1 summarize the analysis carried out and the methods of this study. It was not possible to perform all experiments on all samples, because of the small size of them. The first 5 samples were sacrificed in order to isolate and characterize CSCs: we checked proliferation, self-renewal abilities and positivity for several stem cell markers (Oct-3/4, nestin, CD133), of the isolated cells after one week in culture conditions that favor stem cell growth (data not shown).

## Results

### Conventional chromosome analysis and FISH reveals differences between LG and HG tumors

**Figure 1:**
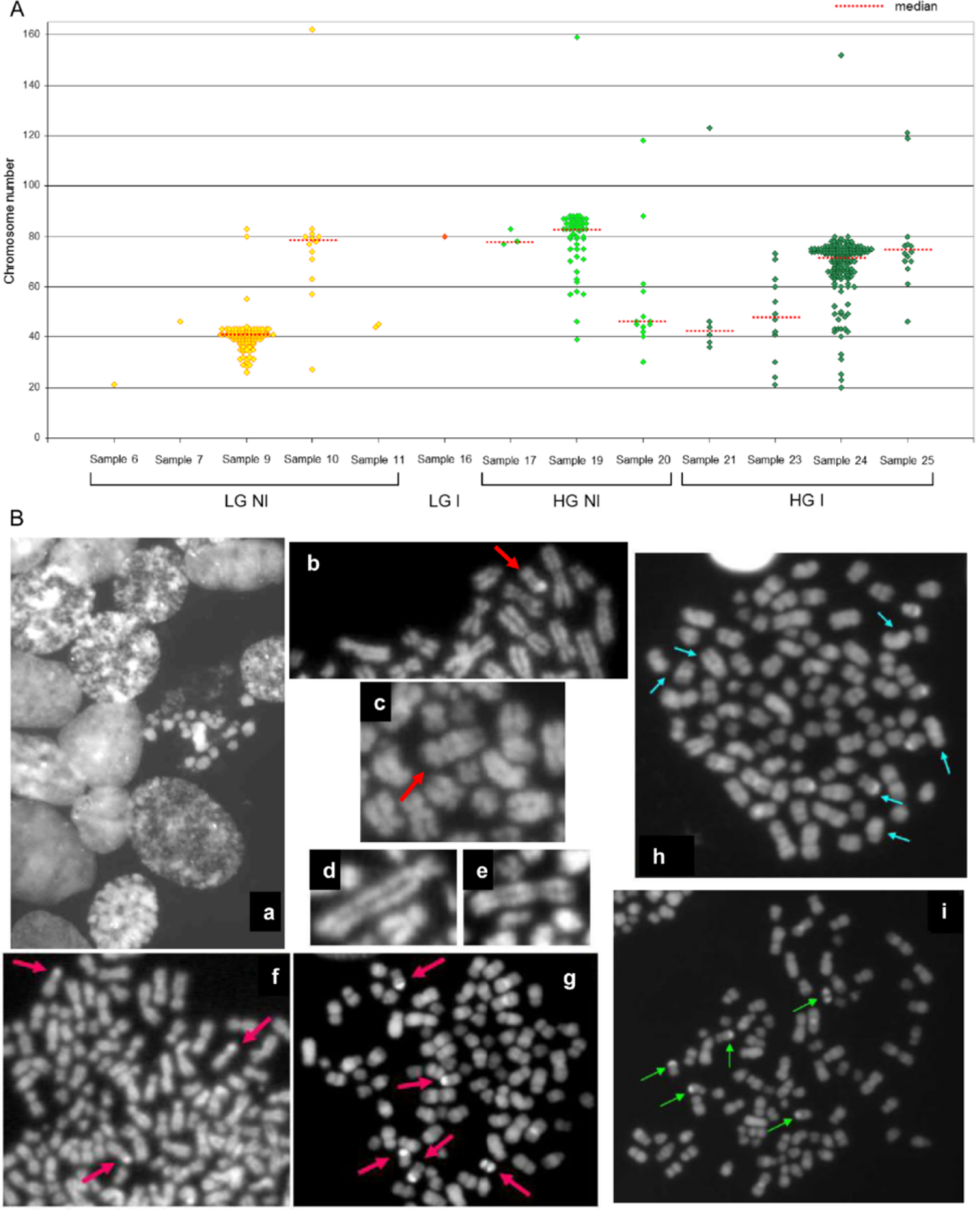
**Conventional chromosome analysis. A)** Distribution of chromosome number per cell in LG and HG samples. **B)** Examples of chromatin and chromosome instability in different metaphases. Chromatin degeneration (a) and chromosome rearrangements: in particular, rearrangements of chromosome Y (b), 12 (c), 4 (d) and 1 (e). Examples of metaphases with chromosome Y rearrangements: clonal rea in the same metaphase (f, arrows) or clonal rea in different metaphases from the same patient (g, i). Example of different rea in the same metaphase (h).

Conventional karyotype analysis was performed on 20 cases, but metaphases were achieved only in 13 cases (65%) (see Table 1). Despite the low number of cases, in 4 out of 6 LG samples most of the cells have a number of chromosomes which ranges from near-haploid (23 ±) to near-diploid (46 ±), while in the remaining samples it ranges from near-triploid (69 ±) to near-tetraploid (92 ±). In HG samples a division in 2 groups was maintained: 3 out of 7 cases have a near-diploid (42.5 - 48) median number of chromosomes, while the remaining cases have a hypertriploid/hypotetraploid (72–83) median number. Moreover, there is a trend towards dispersion in the number of chromosomes per cell, moving from LG to HG (Figure 1A). In addition, evidence for a general chromatin instability and degeneration was observed together with many different numerical and structural aberrations, especially for Y chromosome (Figure 1B).

**Figure 2:**
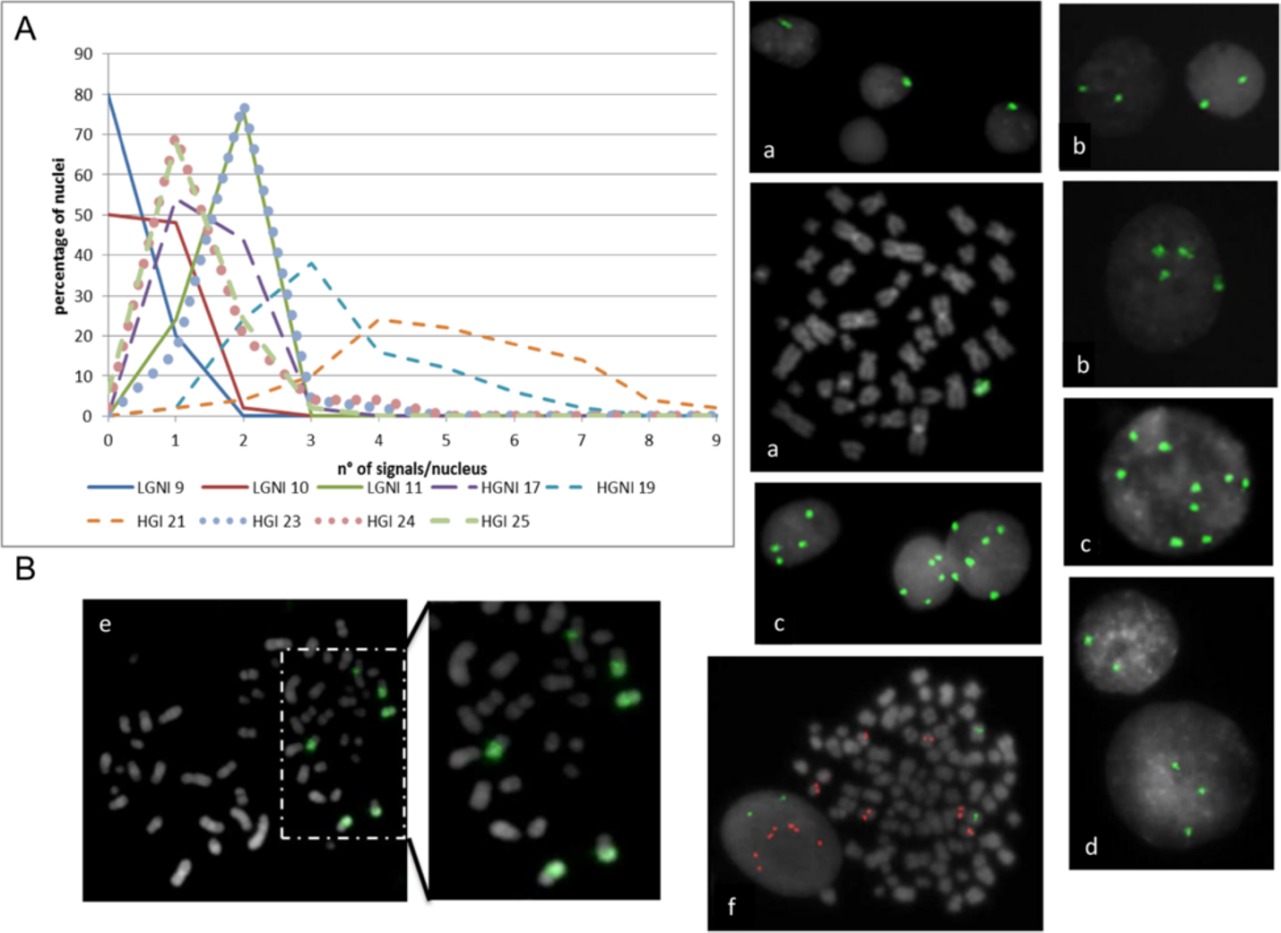
**FISH analysis. A)** Distribution of WCP Y probe signals on interphase nuclei of 9 cases. **B)** Examples of nuclei and metaphases with a correct number of chr Y (a, case 25) and with polysomy of chr Y from different patients (b: case 23, c: case 21, d: case 24). Rearrangements of chr Y highlighted by WCP probe on metaphase (e) and polysomy of chr Y identified by SRY probe (f, red), that is not found for the X chromosome (f, green) in the same patient (case 19).

We have also carried out an in-depth study of the Y chromosome by FISH analysis using the WCPY probe on interphase nuclei of 9 cases. Despite the presence of a great heterogeneity, i.e. the percentage of cells and the number of signals (see the graphic in Figure 2A), we evidenced a trend to the complete loss of Y chromosome in two LG non-invasive cases (9 and 10); three HG samples (17, 24 and 25) manifested the propensity to maintain the Y chromosome, but with a slight trend to acquire one additional copy (more evident for the non-invasive case 17); two overlapped cases (11 and 23), with different grade and stage, show a tendency to two copies of Y chromosome; the last two cases (19 and 21, both HG invasive cases) show a trend to acquire two or even more copies of the chromosome; this propensity is not found for the X chromosome (see Figure 2B).

**Table 1:** **Conventional chromosomal analysis**

Patient	Histotype	N. of metaphases	Range of chr/cell	Median	Modal number	% cells with						
						n±	2n±	3n±	4n±	5n±	6n±	7n±
6	LG NI	1	21			100						
7	LG NI	1	46				100					
11	LG NI	2	44-45				100					
10	LG NI	13	27-162	78	80	7.7	7.7	61.5	15.4			7.7
9	LG NI	93	26-83	41	41	12.9	84.9	1.1	1.1			
16	LG I	1	80					100				
20	HG NI	13	30-118	46	45	7.7	61.5	15.4	7.7	7.7		
17	HG NI	3	77-83	78	ND			66.7	33.3			
19	HG NI	57	39-159	83	83	7	30	61.4			1.8	
21	HG I	6	36-123	42.5	ND		83.3			16.7		
25	HG I	14	46-121	73.5	70		7.1	78.6		14.3		
23	HG I	12	21-73	48	ND	25	41.7	33.3				
24	HG I	152	20-152	72	75	3.3	9.9	86.2				0.6

Summary of conventional karyotype analysis results.

### aCGH analysis on tumor biopsies confirms more altered genomes in HG tumors

We performed aCGH analysis on the subsequent 20 tumor biopsies, 10 HG and 10 LG (see Additional file 1: Table S1), identifying a total of 495 aberrations. As expected, HG tumors were generally more affected than LG: average of 40.3 aberrations per tumor in HG versus 9.2 per tumor in LG (Additional file 3: Table S2). In particular, 120 versus 55 were loss, 241 versus 25 were gain, and 42 versus 12 were amplification. All chromosomes harboured a spectrum of alterations in multiple tumors. The chromosomes that had the fewest aberrations were chromosomes 21 and 18 with 4 and 10 aberrations respectively; most aberrations were found on chromosome 6 (n = 46). Other chromosomes with high aberrations counts were chromosomes 1, 2, 9 and 11 with 37, 38, 34 and 32 alterations respectively. More detailed information on all specific chromosomal regions altered either by copy number gains or losses in tumor biopsies is provided in Additional file 4: Table S4.

### Bladder cancer genomic aberrations: comparison between biopsy samples and the respective CSCs

The most interesting aspect of this work is the comparison of the genomic profiles of 16 biopsies (6 HG and 10 LG) with their respective CSC subpopulations isolated from them (see Material and Methods and Additional file 1: Table S1). We analyzed by aCGH the genomic profiles of CSC subpopulations evidencing a total of 614 aberrations (Additional file 5: Table S3). Surprisingly, HG tumors were less affected than LG tumors as the average of aberrations was 16.83 in HG versus 51.3 in LG. In particular, the largest imbalance between LG and HG is in losses (average 46.6 per tumor LG versus 7 per tumor HG, respectively). The chromosomes that had the most losses were chromosomes 1, 2, 19 with 41, 35 and 35 losses, respectively, in LG tumors. More detailed information on all specific chromosomal regions altered either by copy number gains or losses in CSC subpopulations is provided in Additional file 6: Table S5.

**Figure 3:**
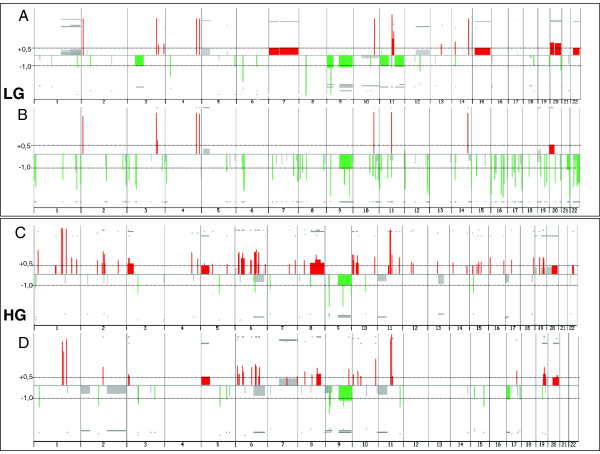
**Summary of copy number analysis (CytoGenomics v2.0 software; Agilent).** Low grade (LG) biopsies **(A)** and their correspondent cell cultures **(B)**; high grade (HG) biopsies **(C)** and their correspondent cell cultures **(D)**. All the samples were analyzed using human 8 x 60 K CGH microarrays (Agilent). The y-axis represents log2 ratio value. The x-axis represents the genomic position of probes with chromosome numbers indicated. Significative gains (log2 ratio > +0.5) and losses (log2ratio < −1.0) are shown in red and green colors, respectively. Gray color represents nonsignificant recurrence of aberrations.

**Figure 4:**
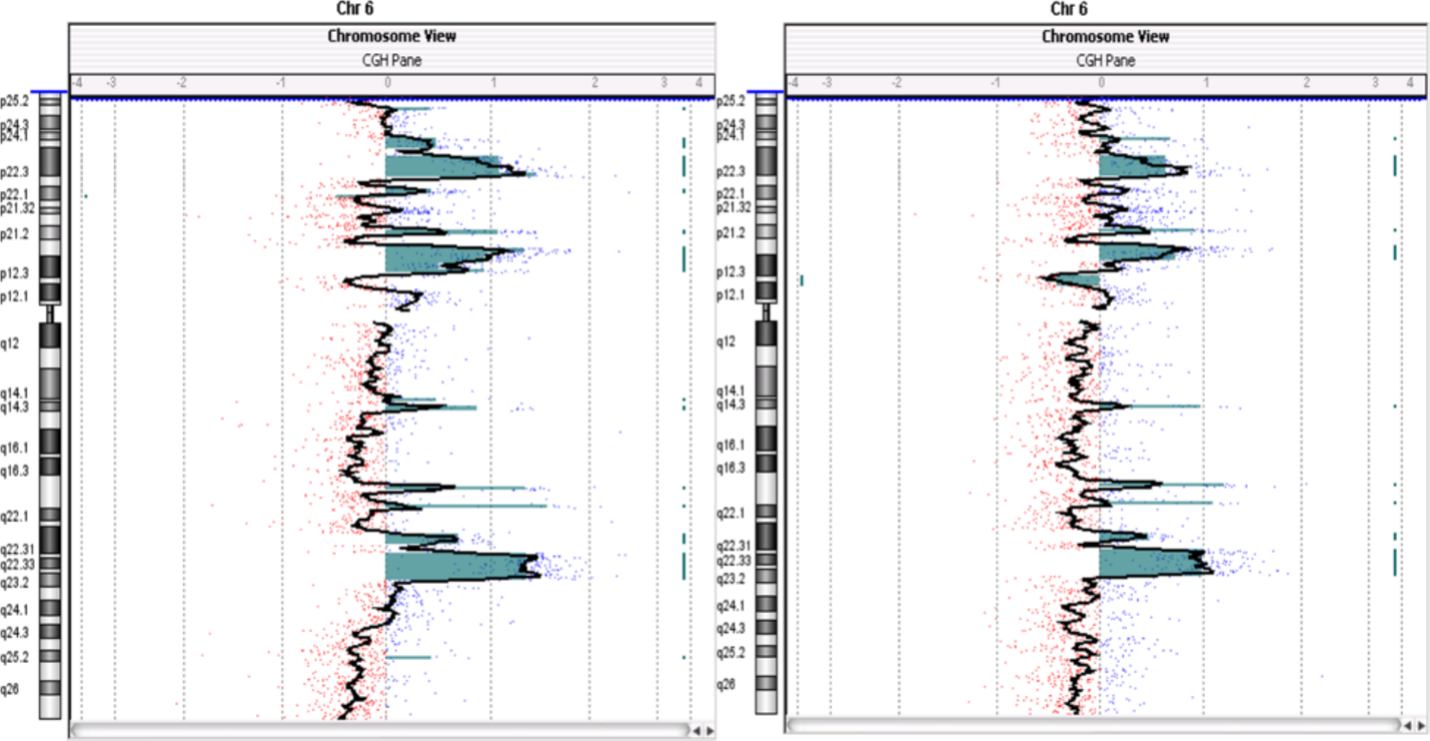
**Example of chromosome 6 chromothripsis both in biopsy (left) and in isolated cells (right) of the same tumor (n. 36).** The x-axis represents log2 ratio value. Significative gains (log2 ratio > +0.5) and losses (log2ratio < −1.0) are shown as coloured regions.

Comparing the overall aberrations per chromosome of biopsies with their respective isolated cells, we found a trend reversal between HG and LG tumors because in HG group the cells have a lower number of CNAs compared to the initial biopsy, while in LG group it is the exact opposite (Figure 3). In order to find shared aberrations between a specific biopsy and the respective isolated cells, we compared the paired genomic profiles (“biopsy” and “isolated cells”) for each sample. Overall, there is a good overlapping between biopsies and isolated cells (see Additional file 7: Table S6 A, B, C). Validation experiment using UroVysion FISH was performed for the most frequent alteration, the loss of 9p21, location of the *P16* tumor suppressor gene, and also the most conserved between biopsies and isolated cells (Additional file 8: Figure S2). Finally, considering the cases with more aberrations, three samples were HG tumors (38, 36, 45) with 18, 13 and 10 aberrations, respectively; among the LG tumors, two samples (27, 35) have 13 and 9 aberrations, respectively. On the basis of the large number of clustered breakpoints at chromosome 6, the chromothripsis hypothesis may be invoked in two tumors (36 and 37) (Additional file 9: Figure S3). The chromothripsis phenomenon is the shattering of two or more chromosomes and their reassembly into derivative chromosomes in a single catastrophic event [28]. Interestingly, the same pattern of alterations were found both in biopsy and in isolated cells of tumor 36, emphasizing the earliness of this event (see Figure 4).

### Gene ontology analysis confirms two different ways in LG and HG tumors

We performed a gene ontology analysis using the GOstat software in order to find statistically over-represented GO terms within groups of genes included in CNAs evidenced by aCGH. Additional file 10: Table S7 reported statistically significant (p < 0.05) GO terms for each sample. The different colors refer to genes contained in gained regions (red), or lost (green), or both (yellow). Two main classes of GO terms are the most represented: *transcription* and *apoptosis*.

For *transcription* a general statistically significant over-representation emerged in biopsies, both in LG than in HG tumors, although in the latters it can be noted a greater presence of red color corresponding to gained genes. As regards CSCs, the over-representation of red (gained genes) was confirmed in HG; conversely in LG samples prevailed the green color (lost genes), except for sample 34. It’s interesting to note that two tumors (27 and 32) showed a GO terms reversal in concomitance with the passage from biopsy to CSCs, from red to green color.

For *apoptosis* emerged a significant over-representation for both gained and lost genes (yellow color) in HG biopsies and in the LG sample 30, while two LG biopsies (27 and 34) were found with over-representation for gained genes and two others (26 and 33) for lost genes. Conversely, there was a clear division in CSCs: six samples (one of which is HG) had a significant over-representation for lost genes, while five tumors (one of which is LG) for gained genes.

For the other ontology classes it was observed a very intricate situation for biopsies, while in CSCs again seems to prevail the green color (lost genes) for LG samples, whereas the red (gained genes) for HG samples.

## Discussion

This study focuses on the complexity of the bladder cancer genome, and for the first time to our knowledge, on the possibility to compare genomic alterations of *in vitro* selected cancer stem-like cells and their original biopsy in order to identify different genomic signature already present in the early stages of tumorigenesis of LG and HG tumors. Despite the low number of cases enrolled and therefore, at the moment, the interpretation may be only hypothetical, the findings of this study may assume a very important significance to those with closely related research interest. Firstly, we performed a conventional karyotype on 20 biopsies (11 LG and 9 HG) in order to delineate the status of ploidy in bulk tumors. Despite this technique is far from being innovative, we observed that LG tumors generally have near diploid metaphases, while HG tumors have a tendency to triploidy with a greater dispersion in the number of chromosomes per cell. Furthermore, we revealed that only two samples out of nine, both non-invasive LG, show a strong tendency to Y chromosome loss. Conversely, the others maintain or, at most, show an opposite tendency to acquire multiple copies of this chromosome. Although the number of cases is very low, this observation is a little in contrast with a recent study showing that Y chromosome losses are equally frequent in urothelial bladder cancer of all grades and stages [29]. Our data seem to agree with another study that showed a significant association of Y polysomies with HG invasive tumors [30]. The overall observations obtained by conventional chromosomal analysis and FISH have confirmed a greater aggressiveness of HG tumors than LG ones; in addition, although a larger number of cases must be studied, we believe that the involvement of the Y chromosome has yet to be clarified and that it is no coincidence that men are more affected by this type of cancer [31].

In this work we present a comprehensive catalog of CNAs across 20 tumor fresh biopsies providing an overview of their common alterations. The overall data evidenced a general chromosomal instability, especially in HG tumors, with a general CNAs ratio of 4:1 respect to LG tumors, and a ratio of 10:1 considering only gains. A previous study by HR-CGH analysis had reached the same conclusion [13]. In addition, in the present work we unveiled an opposite situation analyzing the genomic profiles of CSCs, as in HG tumors they were less affected than in LG tumors, with a general ratio of 1:3 (1:6 if we consider only the losses). To understand this anomalous behavior it would be useful to compare the genomic alterations of the original biopsy with their isolated cells because this approach may focus on the alterations most involved in tumorigenesis of TCCs. The overview of CNAs per chromosome (Figure 3) evidences a better conservation between cells and biopsies of HG tumors than LG tumors, even if isolated cells of HG group are less altered than their original biopsies while the situation is reversed in LG group. In two HG samples the complex pattern of CNAs involving chromosome 6 is consistent with “a chromothripsis like event”. Morrison et al. have recently reported the chromothripsis phenomenon in muscle-invasive TCCs [32]. These authors postulated that chromothripsis is related to a defective replication-licensing complex and that it could lead to intratumoral mutational heterogeneity. However, a recent work suggests that more stringent criteria must be used to identify chromothripsis and that it cannot be distinguished from other complex genomic rearrangements [33]. According to these authors, great caution should be exercised when labeling complex rearrangements as chromothripsis from genome hybridization and sequencing experiments. In our study we identified several shared gain/amplifications in chromosome 6 between the biopsy and the isolated cells of the same tumor, providing evidence in favor of a non-progressive mechanism.

The differences between HG and LG tumors also emerged by GO analysis, especially for two functional GO classes: *transcription* and *apoptosis*. Additional file 10: Table S7 shows a preponderance of GO terms for *transcription* class derived from gained genes of biopsies and isolated cells of HG tumors. Although some LG tumors exhibited a similar behavior (i.e. 34 sample), statistical significance for these tumors is determined by lost genes, especially in isolated cells where it has been shown many lost regions. Similarly, GO terms for *apoptosis* class were derived from lost regions of isolated cells of LG tumors, while the same GO terms were linked to gained regions of isolated cells of HG tumors. Thanks to the comparative analysis between biopsies and CSCs isolated from them, we can speculate that the driving forces of tumorigenesis are quite different in HG and LG tumors, even showing a complementary behavior. Specifically, we confirmed a good correlation between the total number of CNAs and genomic instability with increasing stage and grade of the biopsy [15]. Moreover, we found that CSCs isolated from LG biopsies accumulate a disproportionate number of genomic losses, so the isolated cells would be much altered respect to their original biopsy. This phenomenon was not observed in HG tumors, then it would not seem the result of purely random alterations due to culture conditions, but to essential characteristics which diversify the two types of tumor. Furthermore, GO stat analysis and aCGH data evidenced a subgroup of LG tumors where this paradox seems to be more evident. It would be interesting to verify if this subgroup of LG tumors could have a more aggressive potential and a greater propensity to progress and invade.

## Conclusions

In this study we provide not only an overview of changes in the CNAs of HG vs LG tumors, but for the first time to our knowledge we also make a comparison of the overall CNAs in biopsies and CSCs isolated from them. Our approach indicate that LG differ from HG regarding their respective genomic profile also in the early stage of tumorigenesis; moreover it has been identified a subgroup of LG samples in which the tendency to loss of genomic regions is significantly higher. These findings provide valuable information to deeper study TCC carcinogenesis and may be applicable in the clinic for the identification of patients who will benefit from a more aggressive therapy.

## Electronic supplementary material

Additional file 1: Table S1: Clinic-pathologic characteristics of samples. Histotype, grade and type of analysis are indicated. (DOC 86 KB)

Additional file 2: Figure S1: The two step strategy of analysis applied in this study. (PPT 305 KB)

Additional file 3: Table S2: List of aberrations for each chromosome in 20 tumor biopsies. (DOC 70 KB)

Additional file 4: Table S4: Specific chromosomal regions altered either by copy number gains or losses in tumor biopsies. Chromosomal positions were in accordance with the Human Genome Browser – hg18 assembly (NCBI Build 36.1). (XLSX 82 KB)

Additional file 5: Table S3: List of aberrations for each chromosome in 16 CSC subpopulations. (DOC 67 KB)

Additional file 6: Table S5: Specific chromosomal regions altered either by copy number gains or losses in CSC subpopulations. Chromosomal positions were in accordance with the Human Genome Browser – hg18 assembly (NCBI Build 36.1). (XLSX 155 KB)

Additional file 7: Table S6: Shared aberrations between biopsies and CSC subpopulations. (DOC 176 KB)

Additional file 8: Figure S2: Validation experiment using UroVysion FISH of the most common alteration (9p21 loss) evidenced by aCGH. UroVysion consists of fluorescently labeled DNA probes to the pericentromeric regions of chromosomes 3 (red), 7 (green), and 17 (aqua) and to the 9p21 band (gold) location of the *P16* tumor suppressor gene. A-B) complete loss of gold signals (9p21) in 27 and 38 samples; C) mosaic loss of gold signals in case 39. See [34] for more details about UroVysion FISH. (PPTX 221 KB)

Additional file 9: Figure S3: Chromosome 6 chromothripsis in sample 36 and 37. (PPT 206 KB)

Additional file 10: Table S7: Statistically significant (p < 0.05) GO terms by GOstat software are reported for each sample distinguishing between biopsies (left) and isolated cells (right). The different colors refer to genes contained in gained regions (red), or lost (green), or both (yellow). Two main classes of GO terms are the most represented: *transcription* and *apoptosis*. (PDF 29 KB)

## Abbreviations

TCCs: Transitional cell carcinomas
LG: Low grade
NI: Non-infiltrating
HG: High grade
IN: Muscle invasion
aCGH: Array comparative genomic hybridization
CNAs: Copy number alterations
CSC: Cancer stem cell
FISH: Fluorescence in situ hybridization.
