# Supplementary material for: Chromosomal imbalances in human bladder urothelial carcinoma: similarities and differences between biopsy samples and cancer stem-like cells
Source: BMC Cancer. 2014 Sep 1;14:646. doi: 10.1186/1471-2407-14-646 (PMC4162911; doi:10.1186/1471-2407-14-646)
Supplement: Supplementary file 1 — Additional file 1: Table S1: Clinic-pathologic characteristics of samples. Histotype, grade and type of analysis are indicated. (DOC 86 KB) [file 12885_2014_4827_MOESM1_ESM.doc]

| Patient | Age* | Sex | Histotype | Grade | CSC isolation | Conventional  cytogenetics | Array-CGH  Pre/post isolation | |
| --- | --- | --- | --- | --- | --- | --- | --- | --- |
| 1 | 72 | M | TCC NI | LG | + |  |  |  |
| 2 | 86 | M | TCC NI | LG | + |  |  |  |
| 3 | 83 | M | TCC NI | LG | + |  |  |  |
| 4 | 80 | F | TCC NI | LG | + |  |  |  |
| 5 | nd | M | TCC NI | LG | + |  |  |  |
| 6 | 79 | M | TCC NI | LG | + | + |  |  |
| 7 | 78 | M | TCC NI | LG | + | + |  |  |
| 8 | 70 | M | TCC NI | LG | + | + |  |  |
| 9 | 75 | M | TCC NI | LG | + | + |  |  |
| 10 | 75 | M | TCC NI | LG |  | + |  |  |
| 11 | 70 | M | TCC NI | LG | + | + |  |  |
| 12 | 66 | M | TCC NI | LG |  | + |  |  |
| 13 | 68 | M | TCC NI | LG |  | + |  |  |
| 14 | 87 | M | TCC NI | LG |  | + |  |  |
| 15 | 68 | M | TCC NI | LG |  | + |  |  |
| 16 | 73 | M | TCC I | LG | + | + |  |  |
| 17 | 65 | M | TCC NI | HG | + | + |  |  |
| 18 | 78 | F | TCC NI | HG | + | + |  |  |
| 19 | 87 | M | TCC NI | HG |  | + |  |  |
| 20 | 80 | F | TCC NI | HG |  | + |  |  |
| 21 | nd | M | TCC I | HG |  | + |  |  |
| 22 | 85 | F | TCC I | HG |  | + |  |  |
| 23 | 67 | M | TCC I | HG | + | + |  |  |
| 24 | 85 | M | TCC I | HG | + | + |  |  |
| 25 | 78 | M | TCC I | HG |  | + |  |  |
| 26 | 81 | M | TCC NI | LG | + |  | + | + |
| 27 | 78 | M | TCC NI | LG | + |  | + | + |
| 28 | 58 | F | TCC NI | LG | + |  | + | + |
| 29 | 63 | M | TCC NI | LG | + |  | + | + |
| 30 | 75 | F | TCC NI | LG | + |  | + | + |
| 31 | 67 | M | TCC NI | LG | + |  | + | + |
| 32 | 83 | M | TCC NI | LG | + |  | + | + |
| 33 | 67 | M | TCC NI | LG | + |  | + | + |
| 34 | 60 | M | TCC NI | LG | + |  | + | + |
| 35 | 77 | M | TCC NI | LG | + |  | + | + |
| 36 | 86 | M | TCC | HG | + |  | + | + |
| 37 | 82 | F | TCC I | HG | + |  | + | + |
| 38 | 93 | M | TCC I | HG | + |  | + | + |
| 39 | 85 | M | TCC I | HG | + |  | + | + |
| 40 | 85 | M | TCC I | HG | + |  | + | + |
| 41 | 68 | M | TCC I | HG | + |  | + | + |
| 42 | 68 | M | TCC NI | HG | + |  | + |  |
| 43 | 73 | M | TCC I | HG | + |  | + |  |
| 44 | 67 | M | TCC I | HG | + |  | + |  |
| 45 | 76 | M | TCC I | HG | + |  | + |  |
|  |  |  |  |  | 35 | 20 | 20 | 16 |

*Age at surgery; NI: non-infiltrating (light colors); I: infiltrating (dark colors); HG: high grade (pink shades); LG: low grade (gray shades); +: successful experiment
