# Supplementary material for: Chromosomal imbalances in human bladder urothelial carcinoma: similarities and differences between biopsy samples and cancer stem-like cells
Source: BMC Cancer. 2014 Sep 1;14:646. doi: 10.1186/1471-2407-14-646 (PMC4162911; doi:10.1186/1471-2407-14-646)
Supplement: Supplementary file 2 — Additional file 2: Figure S1: The two step strategy of analysis applied in this study. (PPT 305 KB) [file 12885_2014_4827_MOESM2_ESM.ppt]

## Slide 1
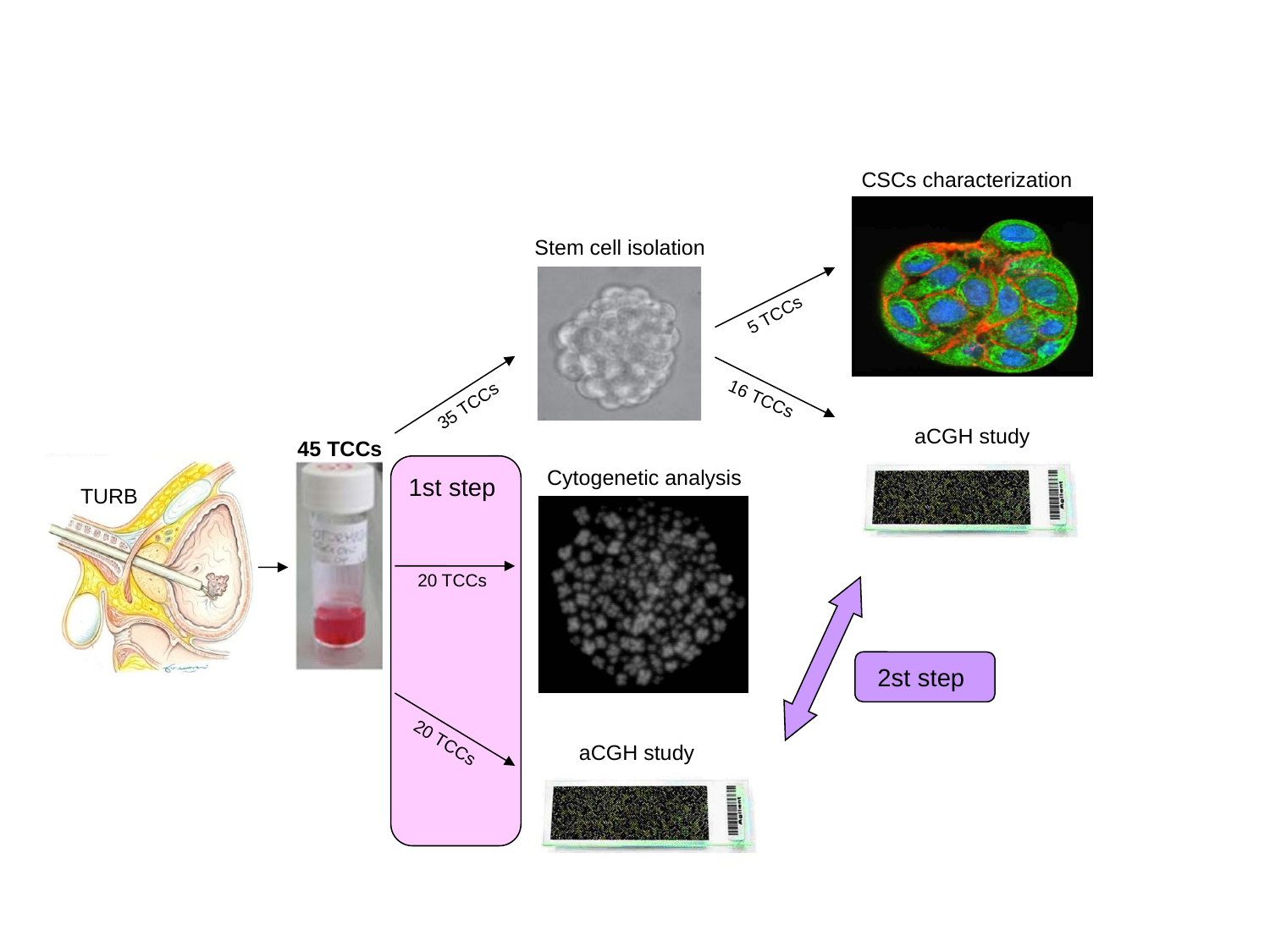

CSCs characterization
Stem cell isolation
5 TCCs
16 TCCs
35 TCCs
aCGH study
45 TCCs
Cytogenetic analysis
1st step
TURB
20 TCCs
2st step
20 TCCs
aCGH study
