# Supplementary material for: Chromosomal imbalances in human bladder urothelial carcinoma: similarities and differences between biopsy samples and cancer stem-like cells
Source: BMC Cancer. 2014 Sep 1;14:646. doi: 10.1186/1471-2407-14-646 (PMC4162911; doi:10.1186/1471-2407-14-646)
Supplement: Supplementary file 3 — Additional file 3: Table S2: List of aberrations for each chromosome in 20 tumor biopsies. (DOC 70 KB) [file 12885_2014_4827_MOESM3_ESM.doc]

**Table S2.** Aberrations patterns in each chromosome in 20 tumor biopsies

| **chrom.** | **Tot. aberr.** | | **Loss** | | **Gain** | | **Amplif.** | |
| --- | --- | --- | --- | --- | --- | --- | --- | --- |
|  | HG n=10 | LG n=10 | HG n=10 | LG n=10 | HG n=10 | LG n=10 | HG n=10 | LG n=10 |
|  | 403 | 92 | 120 | 55 | 241 | 25 | 42 | 12 |
| **1** | 31 | 6 | 7 | 3 | 19 | 3 | 5 | 0 |
| **2** | 30 | 8 | 12 | 4 | 16 | 3 | 2 | 1 |
| **3** | 23 | 4 | 3 | 1 | 17 | 2 | 3 | 1 |
| **4** | 10 | 3 | 4 | 1 | 5 | 0 | 1 | 2 |
| **5** | 14 | 3 | 4 | 2 | 9 | 1 | 1 | 0 |
| **6** | 42 | 4 | 14 | 3 | 21 | 1 | 7 | 0 |
| **7** | 22 | 2 | 2 | 0 | 18 | 2 | 2 | 0 |
| **8** | 25 | 3 | 8 | 3 | 15 | 0 | 2 | 0 |
| **9** | 21 | 13 | 19 | 13 | 2 | 0 | 0 | 0 |
| **10** | 15 | 2 | 2 | 1 | 10 | 0 | 3 | 1 |
| **11** | 20 | 12 | 8 | 6 | 9 | 2 | 3 | 4 |
| **12** | 15 | 2 | 6 | 1 | 6 | 1 | 3 | 0 |
| **13** | 14 | 1 | 4 | 1 | 10 | 0 | 0 | 0 |
| **14** | 10 | 7 | 5 | 4 | 5 | 1 | 0 | 2 |
| **15** | 14 | 2 | 7 | 0 | 7 | 2 | 0 | 0 |
| **16** | 14 | 1 | 5 | 1 | 8 | 0 | 1 | 0 |
| **17** | 15 | 1 | 4 | 1 | 11 | 0 | 0 | 0 |
| **18** | 9 | 1 | 3 | 1 | 6 | 0 | 0 | 0 |
| **19** | 15 | 1 | 0 | 0 | 12 | 1 | 3 | 0 |
| **20** | 15 | 4 | 0 | 1 | 14 | 3 | 1 | 0 |
| **21** | 3 | 1 | 0 | 1 | 3 | 0 | 0 | 0 |
| **22** | 11 | 2 | 5 | 0 | 6 | 2 | 0 | 0 |
| **X** | 12 | 2 | 0 | 0 | 7 | 1 | 5 | 1 |
| **Y** | 3 | 7 | 3 | 7 | 0 | 0 | 0 | 0 |
