# Supplementary material for: Chromosomal imbalances in human bladder urothelial carcinoma: similarities and differences between biopsy samples and cancer stem-like cells
Source: BMC Cancer. 2014 Sep 1;14:646. doi: 10.1186/1471-2407-14-646 (PMC4162911; doi:10.1186/1471-2407-14-646)
Supplement: Supplementary file 5 — Additional file 5: Table S3: List of aberrations for each chromosome in 16 CSC subpopulations. (DOC 67 KB) [file 12885_2014_4827_MOESM5_ESM.doc]

Table S3. Aberrations patterns in each chromosome in 16 CSC subpopulations

| **chrom.** | **Tot. aberr.** | | **Loss** | | **Gain** | | **Amplif.** | |
| --- | --- | --- | --- | --- | --- | --- | --- | --- |
|  | HG n=6 | LG n=10 | HG n=6 | LG n=10 | HG n=6 | LG n=10 | HG n=6 | LG n=10 |
|  | 101 | 513 | 42 | 466 | 48 | 31 | 11 | 16 |
| **1** | 6 | 49 | 0 | 41 | 6 | 8 | 0 | 0 |
| **2** | 7 | 36 | 4 | 35 | 1 | 0 | 2 | 1 |
| **3** | 7 | 23 | 4 | 21 | 3 | 1 | 0 | 1 |
| **4** | 0 | 17 | 0 | 15 | 0 | 0 | 0 | 2 |
| **5** | 4 | 12 | 1 | 12 | 3 | 0 | 0 | 0 |
| **6** | 11 | 20 | 2 | 20 | 3 | 0 | 6 | 0 |
| **7** | 5 | 28 | 1 | 24 | 4 | 4 | 0 | 0 |
| **8** | 8 | 30 | 3 | 29 | 5 | 1 | 0 | 0 |
| **9** | 11 | 24 | 11 | 23 | 0 | 1 | 0 | 0 |
| **10** | 6 | 16 | 1 | 15 | 5 | 0 | 0 | 1 |
| **11** | 8 | 34 | 3 | 28 | 4 | 1 | 1 | 5 |
| **12** | 1 | 26 | 0 | 25 | 1 | 1 | 0 | 0 |
| **13** | 1 | 4 | 1 | 4 | 0 | 0 | 0 | 0 |
| **14** | 3 | 25 | 2 | 22 | 1 | 0 | 0 | 3 |
| **15** | 2 | 18 | 2 | 17 | 0 | 1 | 0 | 0 |
| **16** | 2 | 30 | 1 | 28 | 1 | 2 | 0 | 0 |
| **17** | 5 | 30 | 2 | 28 | 3 | 0 | 0 | 2 |
| **18** | 0 | 8 | 0 | 8 | 0 | 0 | 0 | 0 |
| **19** | 9 | 38 | 2 | 35 | 6 | 3 | 1 | 0 |
| **20** | 2 | 18 | 0 | 15 | 2 | 3 | 0 | 0 |
| **21** | 0 | 5 | 0 | 5 | 0 | 0 | 0 | 0 |
| **22** | 1 | 15 | 1 | 12 | 0 | 2 | 0 | 1 |
| **X** | 1 | 2 | 0 | 1 | 0 | 1 | 1 | 0 |
| **Y** | 1 | 5 | 1 | 3 | 0 | 2 | 0 | 0 |
