# Supplementary material for: Chromosomal imbalances in human bladder urothelial carcinoma: similarities and differences between biopsy samples and cancer stem-like cells
Source: BMC Cancer. 2014 Sep 1;14:646. doi: 10.1186/1471-2407-14-646 (PMC4162911; doi:10.1186/1471-2407-14-646)
Supplement: Supplementary file 7 — Additional file 7: Table S6: Shared aberrations between biopsies and CSC subpopulations. (DOC 176 KB) [file 12885_2014_4827_MOESM7_ESM.doc]

| **Table S6A** | **Losses in sharing** | | | |
| --- | --- | --- | --- | --- |
| **ID tumor** | **Biopsies** | | **CSC subpopulations** | |
|  | **cytoband** | **% of mosaicism ^§^** | **shared cytoband** | **% of mosaicism ^§^** |
| **27 LG** | 1q22 | 40% | 1q21.3-q22 | complete loss |
| **28 LG** | 1q42.13 | 76% | Yes | 94% |
| **29 LG** | 1p22.2-p22.1 | 77% | Yes | 84% |
| **34 LG** | 2p22.3 | 58% **in gain** | Yes | 39% |
| **39 HG** | 2q22.1-37.3 | 26% | Yes | 56% |
| **29 LG** | 3p21.31-p11.1 | 86% | Yes | 74% |
| **36 HG** | 3p14.2 | non mosaic | Yes | 96% |
| **36 HG** | 5q21.1 | non mosaic | Yes | non mosaic |
| **32 LG** | 6p21.31 | complete loss | slightly modified | complete loss |
| **36 HG** | 6p12.3-p12.1 | 38% | Yes | 54% |
| **40 HG** | 6q16.1-q25.3 | 56% | slightly modified | 67% |
| **38 HG** | 7p21.1 | 91% | Yes | 62% |
| **30 LG** | 8p11.23-p11.22 | complete loss | Yes | complete loss |
| **34 LG** | 8p11.23-p11.22 | complete loss | Yes | complete loss |
| **35 LG** | 8p11.23-p11.22 | complete loss | Yes | complete loss |
| **38 HG** | 8q13.2 | 77% | Yes | 47% |
| **40 HG** | 8p22-p21.2 | 39% | slightly modified | 54% |
| **27 LG** | 9p21.3 | complete loss | Yes | complete loss |
|  | 9q13-q34.2 | 87% | slightly modified | 94% |
| **29 LG** | 9p24.3-p13.1 | 90% | shorter | 88% |
|  | 9p21.3 | complete loss | Yes | complete loss |
| **35 LG** | 9p21.3 | complete loss | larger | complete loss |
|  | 9q32.33.1 | non mosaic | Shorter and slitted | complete loss |
| **38 HG** | 9p21.3-p21.1 | non mosaic | Yes | 68% |
|  | 9p21.3-p21.1 | complete loss | Shorter | non mosaic |
|  | 9q13-q34.3 | 44% | slightly modified | 32% |
|  | 9q22.31 | complete loss | Yes | non mosaic |
| **39 HG** | 9p24.3-p13.3 | 29% | Larger | 44% |
|  | 9p21.3 | 68% | slightly modified | non mosaic |
|  | 9q13-q34.3 | 74% | Yes | 84% |
| **40 HG** | 9p24.1-p23 | 39% | slightly modified | 63% |
|  | 9p21.3 | shorter non mosaic | Yes | non mosaic |
|  | 9q13-q34.3 | 57% | Yes | 74% |
| **27 LG** | 10q23.2 | 93% | slightly modified | non mosaic |
| **29 LG** | 11p15.5-p11.12 | 85% | slightly modified | 80% |
|  | 11q14.1-q25 | 86% | Yes | 80% |
| **38 HG** | 11p15.4-p13 | 32% | slightly modified | 27% |
| **40 HG** | 11p15.5-p11.12 | 50% | Yes | 60% |
| **41 HG** | 11q23.3 | 80% | Yes | non mosaic |
| **30 LG** | 12q14.1 | non mosaic | Yes | non mosaic |
| **38 HG** | 13q21.1-q31.2 | 66% | Yes | 32% |
| **31 LG** | 14q24.2 | 28% | Yes | 51% |
| **40 HG** | 14q32.12-q32.13 | 47% | slightly modified | 63% |
| **38 HG** | 15q11.2 | non mosaic | Larger | 73% |
| **39 HG** | 15q11.2 | 46% | Yes | 67% |
| **35 LG** | 16p13.3 | 46% | Shorter | 64% |
| **27 LG** | 17p13.3 | 87% | Larger | non mosaic |
| **40 HG** | 17p13.3-p11.2 | 57% | Yes | 78% |
| **27 LG** | 18q21.32 | 81% | Yes | 70% |
| **32 LG** | 19p13.3-p13.11 | 84% **in gain** | Loss | 64% |
| **27 LG** | 21q22.11-q22.3 | 77% | Larger | non mosaic |
| **38 HG** | 22q11.1-q11.21q | 66% | Slightly modified | 41% |
| **35 LG** | Yp11.31-p11.2 | Complete loss | Yes | Complete loss |
|  | Yq11.21-q11.23 | Non mosaic | Yes | Non mosaic |
| **Total** | LG biopsies = 25 | HG biopsies = 27 | LG CSCs = 27 | HG CSCs = 27 |

**^§^** Estimated percentage of mosaicism by Cheung et al. [26].

| **Table S6B** | **Gains in sharing / mosaicism (%)** | | | |
| --- | --- | --- | --- | --- |
| **ID tumor** | **Biopsies** | | **CSC subpopulations** | |
|  | **cytoband** | **% of mosaicism ^§^** | **shared cytoband** | **% of mosaicism ^§^** |
| **35 LG** | 1q31.2-q44 | 67% | Yes | 90% |
| **39 HG** | 1q21.1-q44 | 55% entire q arm | Yes | 46% |
| **34 LG** | 2p22.3 | 58% | **In loss** | 39% |
| **39 HG** | 2q22.1-37.3 | 26% | Yes | 56% |
| **27 LG** | 3q26.1 | non mosaic | Yes | non mosaic |
| **36 HG** | 3p25.3-p24.3 | non mosaic | slightly modified | non mosaic |
| **38 HG** | 3p16.3-p24.3 | 68% | slightly modified | 73% |
| **38 HG** | 5p15.33-p11 | 55% | Yes | 79% |
| **36 HG** | 6p25.2 | 69% | Yes | 73% |
|  | 6q22.31 | non mosaic | slightly modified | 91% |
| **34 LG** | 7p22.3-p11.1 | 89% | slightly modified | 91% |
|  | 7q11.21-q36.3 | 88% | slightly modified | 93% |
| **40 HG** | 7q11.21-q36.3 | 60% | 7q11.21-q36.2 | 68% |
| **36 HG** | 8p11.23-p11.22 | non mosaic | Yes | non mosaic |
|  | 8q21.11 | non mosaic | slightly modified | non mosaic |
|  | 8q22.1-q24.13 | non mosaic | slightly modified | non mosaic |
| **39 HG** | 8p11.23-p11.22 | non mosaic | Yes | non mosaic |
| **38 HG** | 10q11.21-q11.22 | 84% | Yes | 75% |
| **36 HG** | 12q14.1 | non mosaic | Yes | non mosaic |
| **36 HG** | 14q11.2 | non mosaic | Yes | non mosaic |
| **34 LG** | 15q11.1-q26.3 | 88% | Yes | 91% |
| **36 HG** | 17q12 | non mosaic | Yes | 81% |
| **38 HG** | 17q11.1-q25.3 | 30% | Yes | 30% |
| **32 LG** | 19p13.3-p13.11 | 84% | **In Loss** | 64% |
| **38 HG** | 19p13.3-p13.3 | 73% | Yes | 55% |
|  | 19q12-q13.43 | 68% | slightly modified | 66% |
| **39 HG** | 19p13.3-p12 | 25% | Shorter | 64% |
|  | 19q12-q13.43 | 25% | Slightly modified | 45% |
| **34 LG** | 20p13-p11.1 | 71% | Yes | 81% |
|  | 20q11.21-q13.33 | 63% | Yes | 92% |
| **38 HG** | 20p13-q13.33 | 87% | Yes | 66% |
| **34 LG** | 22q11.1-q13.33 | 81% | Yes | Non mosaic |
| **30 LG** | Xp11.4 | 99% | Yes | 95% |
| **Total** | LG biopsies = 11 | HG biopsies = 22 | LG CSCs = 9 | HG CSCs = 22 |

**^§^** Estimated percentage of mosaicism by Cheung et al. [26].

| **Table S6C** | **Amplifications in sharing** | | | |
| --- | --- | --- | --- | --- |
| **ID tumor** | **Biopsies** | | **CSC subpopulations** | |
|  | **cytoband** | | **shared cytoband** | |
| **38 HG** | 1q21.2 | | Yes | |
|  | 1q21.3 | | Yes | |
|  | 1q25.1 | | Yes | |
|  | 1q32.1 | | NO | |
| **41 HG** | 1p36.12 | | Log2 ratio= 0.86 | |
| **35 LG** | NO | | 1p34.2 | |
| **27 LG** | 2p25.1 | | Yes | |
| **36 HG** | 2q14.1-q14.3 | | NO | |
|  | 2q14.1-q14.3 (inside) | | Yes (slightly modified) | |
| **40 HG** | Log2 ratio= 0.96 | | 2p11.2 | |
| **27 LG** | 3q25.2 | | Yes | |
| **27 LG** | 4q32.2 | | Yes | |
|  | 4q34.3 | | Yes | |
| **40 HG** | 4q31.22 | | NO | |
| **36 HG** | 6p22.3 | | Gain non mosaic included | |
|  | 6p21.31-p21.2 | | Yes | |
|  | 6p21.1 | | Gain modified non mosaic | |
|  | 6q21 | | Yes | |
|  | 6q21 | | Yes | |
|  | 6q22.31-q23.2 | | Yes | |
|  | Log2 ratio= 0.9 | | 6q14.3 | |
| **40 HG** | NO | | 6p24.3 | |
| **36 HG** | 8q22.2-q22.3 | | NO | |
| **27 LG** | 10q25.1 | | Yes | |
| **38 HG** | 10q26.12-q26.13 | | Yes | |
| **41 HG** | 10p15.1-p14 | | Gain non mosaic | |
|  | 10p11.23-p11.22 | | Gain modified non mosaic | |
| **27 LG** | 11q13.2-q13.3 | | Yes | |
| **29 LG** | 11q13.2-14.1 | | Yes | |
|  | 11q13.2-13.3 (inside) | | Yes | |
|  | 11q14.1 (inside) | | NO | |
|  | NO | | 11q23.3 | |
| **38 HG** | 11q13.2-q13.3 | | Yes | |
| **41 HG** | 11q14.1 | | NO | |
| **41 HG** | 12q14.1 | | NO | |
| **27 LG** | 14q31.3-q32.11 | | Yes | |
| **29 LG** | 14q11.2 | | Yes | |
| **35 LG** | NO | | 14q32.33 | |
| **38 HG** | 16p13.13 | | Gain 58% mosaic | |
| **34 LG** | NO | | 17q21.2 | |
| **35 LG** | NO | | 17q25.3 | |
| **36 HG** | 19q13.13-q13.2 | | Yes slightly modified | |
| **41 HG** | 19p12 | | NO | |
| **37 HG** | 20q11.21 | | NO | |
| **26 LG** | NO | | 22q13.33 | |
| **35 LG** | Xp11.22-p11.21 | | NO | |
| **37 HG** | Xp22.33-p21.1 | | NO | |
|  | Xq25 | | NO | |
|  | Xq26.3-q27.1 | | NO | |
|  | Xq27.1 | | NO | |
| **38 HG** | Xq22.3 | | Yes | |
| **Total** | LG biopsies = 12 | HG biopsies = 31 | LG CSCs = 16 | HG CSCs = 15 |
