# Supplementary figures and images for: Chromosomal imbalances in human bladder urothelial carcinoma: similarities and differences between biopsy samples and cancer stem-like cells
Source: BMC Cancer. 2014 Sep 1;14:646. doi: 10.1186/1471-2407-14-646 (PMC4162911; doi:10.1186/1471-2407-14-646)

## Slide 1
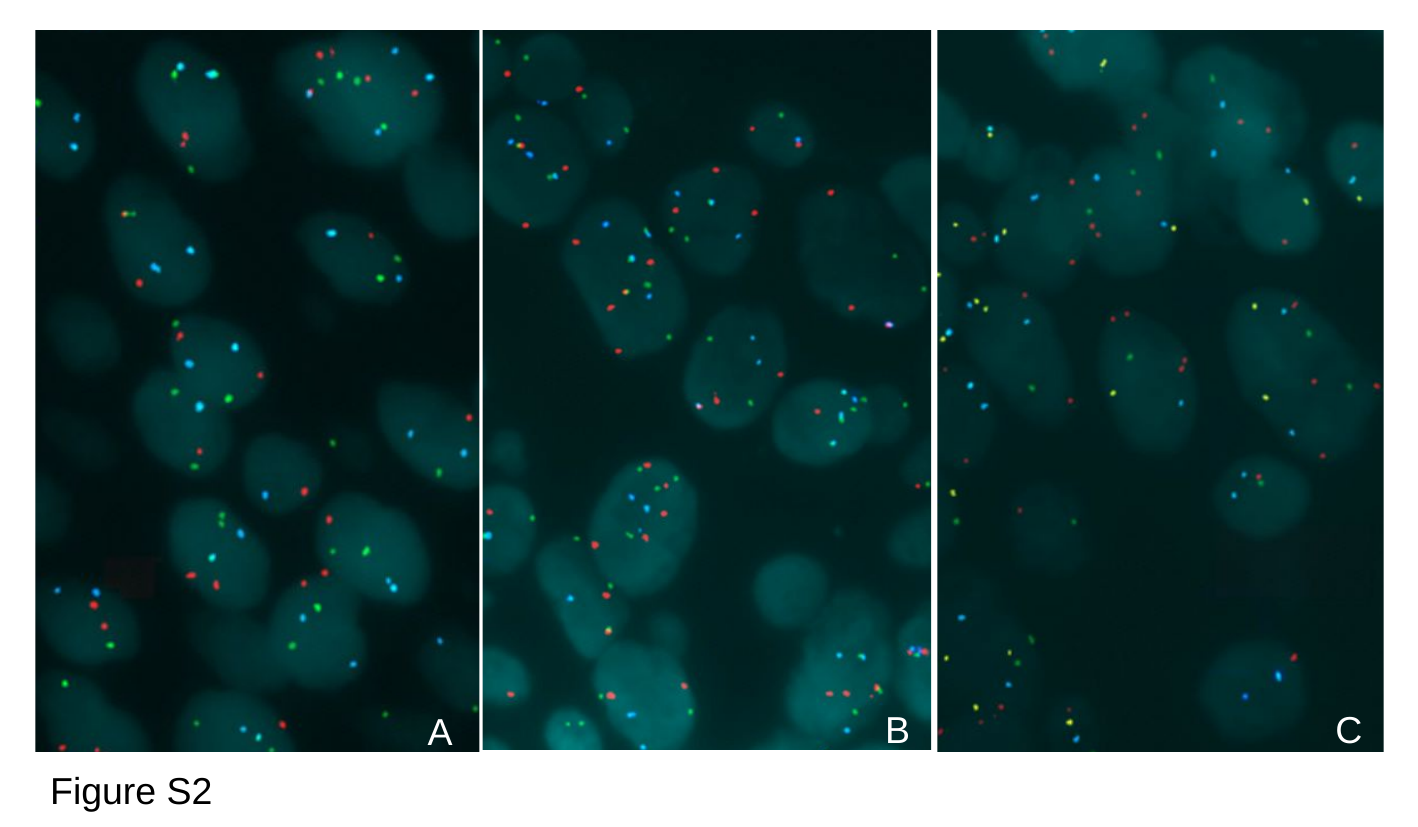

#
B
C
A
Figure S2

Supplement: Supplementary file 8 — Additional file 8: Figure S2: Validation experiment using UroVysion FISH of the most common alteration (9p21 loss) evidenced by aCGH. UroVysion consists of fluorescently labeled DNA probes to the pericentromeric regions of chromosomes 3 (red), 7 (green), and 17 (aqua) and to the 9p21 band (gold) location of the P16 tumor suppressor gene. A-B) complete loss of gold signals (9p21) in 27 and 38 samples; C) mosaic loss of gold signals in case 39. See [34] for more details about UroVysion FISH. (PPTX 221 KB) [file 12885_2014_4827_MOESM8_ESM.pptx]

## Slide 1
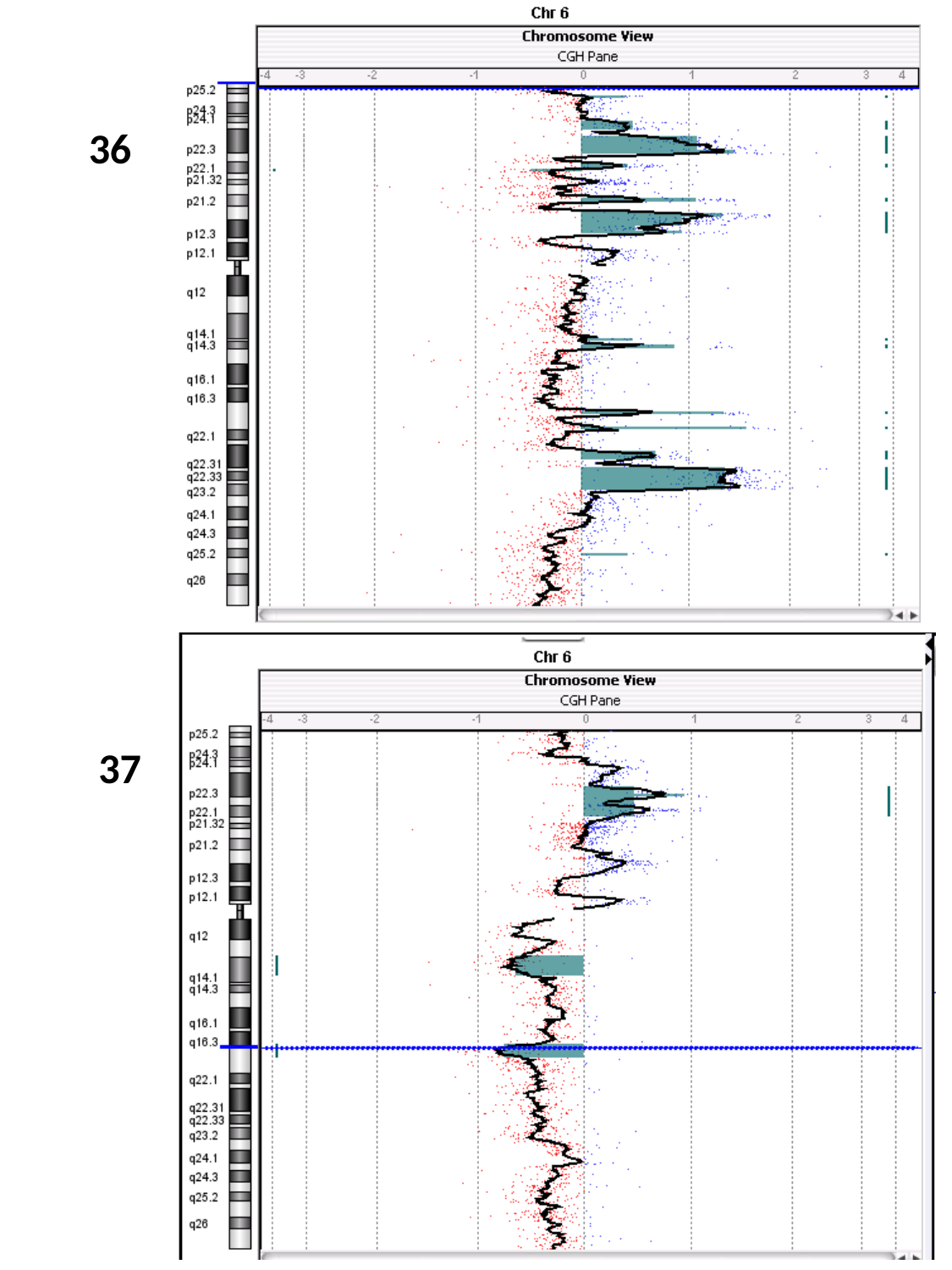

36
37

Supplement: Supplementary file 9 — Additional file 9: Figure S3: Chromosome 6 chromothripsis in sample 36 and 37. (PPT 206 KB) [file 12885_2014_4827_MOESM9_ESM.ppt]
